# Impact of Vaccines Across the Lifespan: A New Perspective in Public Health—Conclusions of an Expert Panel—Part 2 †

**DOI:** 10.3390/vaccines14030204

**Published:** 2026-02-25

**Authors:** Roberto Debbag, María L. Ávila-Agüero, José Brea, Carlos Espinal, Rodrigo Romero-Feregrino, Jaime R. Torres, Hebe Vázquez, Robinson Cuadros, Gustavo Lazo-Páez, Andrea Schilling, Pablo Bonvehí, Maisa Kairalla, Alfonso J. Rodríguez-Morales

**Affiliations:** 1 Latin-American Vaccinology Society, Buenos Aires C1425AWK, Argentina; rdebbag@hotmail.com (R.D.); drbrea@hotmail.com (J.B.); 2 Pediatric Infectious Diseases Department, Hospital Nacional de Niños, San Jose 111221, Costa Rica; avilaaguero@gmail.com; 3 Center for Infectious Disease Modeling and Analysis, Yale University School of Public Health, New Haven, CT 06510, USA; 4 Facultad de Ciencias de la Salud, Instituto Tecnológico de Santo Domingo, Santo Domingo 10602, Dominican Republic; 5 Department of Global Health, Robert Stempel College of Public Health and Social Work, Florida International University, Miami, FL 33199, USA; caespina@fiu.edu; 6 Asociación Mexicana de Vacunología, Instituto Para el Desarrollo Integral de la Salud (IDISA), Instituto Mexicano del Seguro Social (IMSS), CONCAMIN, Saint Luke School of Medicine, Academia Mexicana de Pediatria, Av. Cuauhtémoc 271, Interior 101, Colonia Roma, Cuauhtémoc, Mexico City ZC 06700, Mexico; drrodrigo@idisalud.com; 7 Infectious Diseases Section, Tropical Medicine Institute, Universidad Central de Venezuela, Caracas 1050, Venezuela; jaimerafael.torres@gmail.com; 8 Grupo de Vacunas de la Fundación Centro de Estudios Infectológicos (FUNCEI), Buenos Aires C1425AWK, Argentina; hebevazquez@gmail.com; 9 Asociación Internacional de Gerontología y Geriatría, Comité Latinoamericano y del Caribe, Carrera 7C Bis 139-17, Bogotá 110121, Colombia; robinsoncuadros@gmail.com; 10 Universidad de Ciencias Médicas, San Jose 2060, Costa Rica; gustavo.lazo@gmail.com; 11 Servicio de Inmunología y Reumatología Pediátrica, Hospital Clínica Bíblica, San Jose 10104, Costa Rica; 12 Institute of Science and Innovation in Medicine (ICIM), Facultad de Medicina, Clínica Alemana, Universidad del Desarrollo, Santiago 7610658, Chile; dra.andrea.schilling@gmail.com; 13 Centro de Educación Médica e Investigaciones Clínicas “Norberto Quirno” (CEMIC), Buenos Aires 1430, Argentina; pablobonvehi@gmail.com; 14 Universidade Federal de São Paulo, São Paulo 04021-001, Brazil; maisakairalla@uol.com.br; 15 Faculty of Health Sciences, Universidad Científica del Sur, Lima 15067, Peru; 16 Grupo de Investigación Biomedicina, Faculty of Medicine, Fundación Universitaria Autónoma de las Américas-Institución Universitaria Visión de las Américas, Pereira 660003, Colombia

**Keywords:** life-course vaccination, early-life vaccination, cancer prevention, cardiovascular protection, chronic disease comorbidities, healthy longevity, vaccination policy

## Abstract

This second part of this expert panel review explores the broader impact of vaccines across the lifespan, emphasizing their role beyond immediate infection control. Vaccines not only extend life expectancy but also influence long-term health trajectories, particularly during the critical first 1000 days of life, where they prevent severe infections, disability, and mortality. They contribute to cancer prevention through vaccination against human papillomavirus and hepatitis B, reduce cardiovascular events and complications in individuals with chronic diseases, and protect against late-life functional decline. Furthermore, vaccines exert lasting effects on healthy aging by modulating inflammation and preserving independence, while proposed vaccination schemes illustrate the need for a comprehensive, life-course approach. Together with Part 1, which focused on immunosenescence and immune modulation, this closing installment underscores vaccination as a cornerstone of sustainable health policy, reinforcing its pivotal role in extending both lifespan and healthspan for future generations.

## 1. Introduction

**Figure 1:**
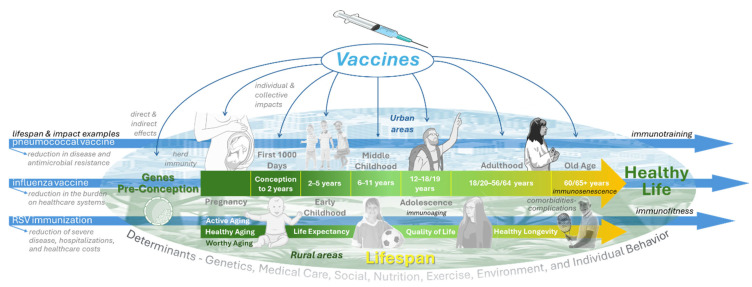
Life-course vaccination and its contributions to healthy aging, disease prevention, and longevity. This figure illustrates the cumulative biological, clinical, and societal pathways through which vaccination across infancy, adolescence, adulthood, and older age reduces disease burden, preserves functional capacity, and extends healthspan.

Vaccination represents one of the most powerful public health interventions, extending its benefits far beyond the prevention of acute infectious diseases [1]. In this second part of our review (see part 1 at https://doi.org/10.3390/vaccines14020183), we focus on the broader life-course impact of vaccines and their capacity to extend both lifespan and healthspan. Figure 1 summarizes the main biological, clinical, and public health pathways through which vaccination influences long-term health trajectories across different life stages.

This conceptual framework highlights how early-life, adult, and older-age vaccination contribute cumulatively to disease prevention, functional preservation, and resilience against age-related decline.

From the very beginning of life, vaccines play a critical role in shaping health trajectories. The first 1000 days, spanning from conception to early childhood, represent a window of exceptional vulnerability and opportunity. Vaccination during this period not only prevents life-threatening infections but also reduces long-term risks of severe disease, disability, and developmental impairment [2]. Similarly, vaccines against human papillomavirus (HPV) and hepatitis B virus exemplify how vaccination contributes to cancer prevention, while emerging evidence links vaccines to reduced cardiovascular events and protection against other chronic comorbidities [3].

Vaccines also exert late effects that influence health in adulthood and old age, underscoring their capacity to modify disease risk well beyond the immediate period of administration [4,5]. This expanding understanding reinforces the need for comprehensive, adaptive, and lifelong vaccination schemes that integrate pediatric, adolescent, adult, and older adult vaccination into a coherent continuum of care [6].

By examining vaccines that extend life, protect during the critical first 1000 days, prevent severe infectious and chronic diseases, and promote long-term resilience, this second part of our review advances the argument that vaccination is not only a medical intervention but also a strategy for sustainable health across generations [7,8].

These challenges and opportunities are particularly relevant in the Americas, where rapid population aging, persistent inequities, and heterogeneous vaccination coverage coexist.

Although this review adopts a global life-course perspective, particular attention is given to the Americas, reflecting the panel members’ expertise, the regional epidemiological context, and data availability. Where possible, findings from other regions are incorporated to provide broader international relevance. This work was developed under the coordination of the Latin American Society for Vaccinology (SLV), which convened an expert panel from multiple countries of the region to develop it, discuss it and present it at an ad hoc meeting in Bogota, Colombia, 26–27 June 2025, and later in the Latin American Symposium on Maternal Immunization of the SLV, Santo Domingo, Dominican Republic, 9–10 December 2025, in addition to virtual meetings and coordinations. We searched studies and articles in multiple databases, including Web of Science, Scopus, PubMed, SciELO, and ScienceDirect. We were supported by an evidence assessment using the OpenEvidence and VeraHealth platforms; however, this is not a systematic or scoping review.

## 2. Vaccines That Extend Life

Vaccines are among the few medical interventions clearly associated with gains in lifespan and with potential benefits for healthspan (Figure 1). By preventing fatal infections and reducing the long-term burden of disease, they contribute to sustained gains in survival across populations [9,10]. Historical and contemporary data show that widespread vaccination against diseases such as smallpox, measles, pertussis, influenza, and pneumococcal infections has added years to average life expectancy [11,12,13,14,15]. Beyond immediate protection, vaccines also reduce complications that accelerate aging, frailty, and the progression of chronic diseases [16,17]. Thus, vaccination must be understood not only as a means of infection control but also as a cornerstone of longevity and healthy aging strategies [18].

### 2.1. Impact of Vaccines in the 1000 Days of Life

The first 1000 days of life, spanning from conception through the first two years, constitute a critical window that shapes survival, growth, and long-term health [19]. During this period, rapid development of the brain and immune system occurs alongside heightened vulnerability to infections, nutritional deficits, and adverse environmental exposures [20]. In low- and middle-income countries, these challenges translate into high rates of maternal and infant mortality, significant morbidity, and long-term health consequences. Preventive strategies during this window are therefore pivotal, and vaccination stands as one of the most effective and impactful interventions [19,20,21,22].

#### 2.1.1. Vaccination as a Pillar of Early Survival

Since the inception of the Expanded Programme on Immunization (EPI) more than five decades ago, vaccines have prevented an estimated 154 million deaths globally, with the majority of lives saved among children under five years of age [22,23,24]. This represents approximately 40% of the global reduction in child mortality observed in recent decades. Each life saved through vaccination yields decades of healthy life, underscoring vaccination as both a survival intervention and an investment in human capital [25].

Vaccination during pregnancy further enhances this impact by conferring passive protection to the newborn via transplacental antibody transfer [26]. Pertussis, influenza, and COVID-19 [27,28], and, more recently, respiratory syncytial virus (RSV) vaccines administered to mothers protect both mother and infant [29,30], substantially reducing the burden of early-life infections [31]. Evidence indicates that maternal Tdap vaccination prevents 70–90% of pertussis cases in newborns, whereas influenza vaccination during pregnancy can reduce influenza illness in infants by more than 60%. RSV prevention through maternal vaccination or monoclonal antibodies has also proven highly effective in reducing severe respiratory disease and mortality in the neonatal period [32,33].

#### 2.1.2. Infant Vaccination and the Transformation of Child Health

Routine vaccination during the first two years of life has dramatically altered the epidemiology of pediatric diseases. The introduction of vaccines against poliovirus, diphtheria, pertussis, *Haemophilus influenzae* type b (Hib), hepatitis B, pneumococcus, meningococcus, rotavirus, and measles has led to steep declines in infant morbidity and mortality worldwide [12]. In many regions, diseases once endemic and deadly have been eliminated or nearly eradicated. For example, Hib vaccination has virtually eliminated bacterial meningitis caused by this pathogen, preventing lifelong disabilities such as deafness and cognitive impairment [34,35]. Hepatitis B vaccination at birth has prevented millions of chronic infections and significantly reduced the incidence of hepatocellular carcinoma in childhood, as documented in Taiwan [36,37]. Likewise, the eradication of polio in most parts of the world has spared generations from paralysis and disability [38].

Despite these successes, setbacks threaten progress. Coverage gaps, exacerbated by health system disruptions during the COVID-19 pandemic, left over 25 million children under-vaccinated in 2021 [39,40]. This has created conditions for the resurgence of measles, polio, and diphtheria in some regions, highlighting the fragility of vaccination achievements and the need to reinforce trust and access to vaccines [41].

#### 2.1.3. Long-Term Benefits Across the Life Course

The benefits of vaccination during the first 1000 days extend far beyond childhood. By preventing infections and their sequelae, vaccines reduce the risk of chronic disease, disability, and premature mortality in adulthood. For instance, avoiding neonatal hepatitis B infection reduces the lifetime risk of cirrhosis and liver cancer [42,43]. Preventing severe respiratory infections in infancy preserves lung function, reducing the likelihood of chronic respiratory diseases later in life [44]. Similarly, eliminating measles not only prevents immediate illness but also protects against the long-term immunosuppression that predisposes children to other infections for years after recovery [45].

In addition, vaccines lower systemic inflammation, protecting developing organs from damage that could predispose individuals to cardiovascular, renal, or metabolic disorders in later life. These mechanisms demonstrate that early-life vaccination contributes to healthier trajectories across decades, directly supporting the concept of vaccines as investments in lifelong health and longevity [46,47,48].

#### 2.1.4. Population and Societal Impact

At the population level, early-life vaccination has been central to increases in life expectancy. By preventing early childhood mortality and disabling sequelae, vaccines support educational attainment, productivity, and economic development. Cohort studies show that vaccinated children are more likely to reach adulthood and contribute productively to society, making vaccination a cornerstone of both public health and sustainable development [49].

The first 1000 days of life are critical for long-term individual and population health. Vaccination during this period protects survival, prevents severe infections and disability, and shapes healthier life-course trajectories. By protecting mothers and infants, early vaccination builds resilience against immediate threats and influences lifelong health. Sustaining these benefits requires high coverage, equitable access, and public trust, positioning early-life vaccination as a strategic investment in healthy aging, societal resilience, and intergenerational well-being [50].

### 2.2. Prevention of Severe Disease

Vaccination is one of the most effective public health strategies to prevent severe disease, disability, and death across the lifespan (Figure 1). Beyond preventing infection, vaccines reduce complications, hospitalizations, and long-term sequelae. Influenza, pneumococcal disease, and herpes zoster disproportionately affect adults and older populations, and their prevention through vaccination protects individual health, reduces healthcare burden, preserves functional capacity, and supports healthy aging [51,52].

#### 2.2.1. Influenza: Preventing Severe Respiratory and Systemic Outcomes

Influenza is a significant cause of seasonal morbidity and mortality, particularly among older adults, pregnant women, young children, and individuals with chronic conditions. Annually, it results in millions of hospitalizations and hundreds of thousands of deaths worldwide, with severe cases often complicated by pneumonia, sepsis, cardiovascular events, and exacerbation of underlying comorbidities [53].

Influenza vaccination reduces severe outcomes, including intensive care admission and death, despite seasonal variability in effectiveness. It is also associated with fewer major cardiovascular events, especially in individuals with heart disease, likely by reducing systemic inflammation and preventing atherosclerotic plaque destabilization [54].

Influenza vaccines provide benefits across age groups. In pregnant women, vaccination protects both mothers and newborns through passive immunity, whereas in older adults, high-dose, adjuvanted formulations enhance responses despite immunosenescence [55,56,57]. Consequently, annual influenza vaccination remains a cornerstone of strategies to reduce global disease burden [55].

#### 2.2.2. Pneumococcal Disease: Preventing Pneumonia, Invasive Infections, and Complications

*Streptococcus pneumoniae* is a leading cause of community-acquired pneumonia, invasive disease, and death among adults, particularly in those over 60 years and in patients with comorbidities such as chronic respiratory or cardiovascular conditions [58,59]. Despite widespread pediatric vaccination programs, significant disease burden persists in adults due to circulating serotypes that are not fully covered by childhood vaccination [60].

Pneumococcal vaccination protects against pneumonia and invasive disease while reducing systemic complications. Severe pneumococcal infections are linked to acute cardiovascular events, such as myocardial infarction and stroke, likely driven by inflammation, platelet activation, endothelial dysfunction, and possible molecular mimicry that may accelerate atherosclerosis [61].

By preventing pneumococcal infections, vaccines reduce not only respiratory disease but also extrapulmonary complications and associated mortality. Recent higher-valent pneumococcal conjugate vaccines, such as PCV20 and PCV21, represent important advances in adult pneumococcal prevention [62,63,64,65]. PCV20 expands coverage beyond PCV13 by incorporating seven additional serotypes associated with residual invasive pneumococcal disease in adults. PCV21 further extends serotype coverage, targeting additional strains associated with the ongoing disease burden in older populations [62,63,64,65]. This was specifically developed for adult use and includes serotypes selected based on their epidemiological relevance in this age group [66]. These vaccines have demonstrated robust immunogenicity in clinical trials, including among older adults in whom immunosenescence may attenuate vaccine responses [66,67,68]. By broadening serotype coverage and enabling simplified vaccination schedules, these newer formulations may enhance protection against invasive disease and pneumococcal pneumonia [69]. Adult pneumococcal vaccination has been associated with reductions in hospitalization, preservation of functional status, decreased antibiotic use, and potential contributions to antimicrobial resistance control [70].

#### 2.2.3. Herpes Zoster: Preventing Chronic Pain and Disability

Herpes zoster (shingles), caused by the reactivation of latent varicella-zoster virus, affects up to one-third of the population during their lifetime, with incidence rising sharply after age 50. The most feared complication, post-herpetic neuralgia (PHN), results in chronic pain that may persist for months or years, profoundly impairing quality of life. Other severe outcomes include ocular involvement leading to vision loss, neurological complications such as meningitis or stroke, and disseminated disease in immunocompromised individuals [71].

The recombinant zoster vaccine (RZV) has significantly modified the prevention of zoster. Unlike the live-attenuated vaccine, RZV provides durable, high efficacy—above 85% across all age groups, including older adults and immunocompromised patients (Table 1). By overcoming immune senescence through a potent adjuvant system, RZV elicits robust humoral and cellular immune responses, thereby ensuring long-lasting protection [72].

To contextualize the clinical performance of the recombinant zoster vaccine across age groups and high-risk populations, Table 1 summarizes efficacy estimates against herpes zoster and post-herpetic neuralgia derived from pivotal randomized controlled trials and subgroup analyses.

As shown in Table 1, the recombinant zoster vaccine demonstrates consistently high efficacy against both herpes zoster and post-herpetic neuralgia across older age groups and immunocompromised populations. Notably, protection remains robust in adults aged ≥70 years and in individuals with underlying conditions, populations traditionally characterized by reduced vaccine responsiveness. These findings highlight the capacity of adjuvanted subunit vaccines to overcome key aspects of immunosenescence and support their prioritization in adult and geriatric immunization programs [73,74,75,76,77,78,79,80].

Beyond preventing acute shingles, vaccination significantly reduces the incidence of PHN, thereby preserving physical functioning and quality of life in vulnerable populations. Table 2 summarizes key patient-centered and health system outcomes associated with RZV across selected high-risk groups. Clinical trials have shown an approximately 90% reduction in PHN incidence, fewer severe pain episodes, and faster symptom resolution in breakthrough cases. Psychological benefits include reduced anxiety and depression associated with shingles, while socioeconomic benefits include fewer missed workdays, reduced caregiver burden, and decreased healthcare costs. Vaccination against herpes zoster is therefore an essential intervention for healthy aging and maintaining independence in older adults [81].

As summarized in Table 2, RZV vaccination is associated not only with reduced disease incidence but also with meaningful improvements in functional status, fewer hospitalizations, and a lower pain burden. These patient-centered outcomes are particularly relevant in older and immunocompromised populations, in whom herpes zoster can accelerate functional decline and dependency. Together, these findings reinforce the role of zoster vaccination in promoting healthy aging and preserving independence.

#### 2.2.4. Integrating Vaccination into Strategies for Preventing Severe Disease

The examples of influenza, pneumococcal disease, and herpes zoster illustrate how vaccines extend beyond infection prevention to avert severe complications, disability, and premature death. They reduce systemic inflammation, protect against secondary cardiovascular and neurological outcomes, and mitigate long-term sequelae. Significantly, their benefits extend to both the individual and population levels: preserving functional ability, reducing strain on the healthcare system, and supporting economic productivity [52,82].

However, challenges remain. Vaccine uptake in adults is suboptimal worldwide, particularly in low- and middle-income countries where access is limited, and awareness is low. Overcoming these barriers requires policies that prioritize adult vaccination, integrate vaccination into chronic disease management, and address vaccine hesitancy. In the context of aging populations and increasing comorbidity burdens, the role of vaccines in preventing severe disease must be recognized as central to strategies for promoting longevity and healthy aging [83].

### 2.3. Cancer Prevention

**Figure 2:**
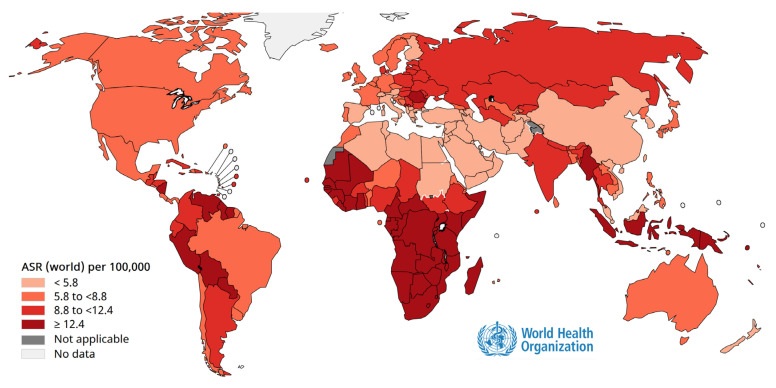
Global burden of HPV-attributable cancers and implications for vaccination-based prevention. Age-standardized incidence rates of cancers attributable to human papillomavirus worldwide in 2020, according to the International Agency for Research on Cancer (IARC–WHO). The figure highlights substantial regional disparities and the potential impact of high-coverage HPV vaccination programs. Source: IARC Global Cancer Observatory: https://gco.iarc.who.int/causes/infections/tools-map?mode=1&sex=0&continent=0&agent=8&cancer=0&key=asr&scale=quantile (accessed on 1 December 2025).

**Figure 3:**
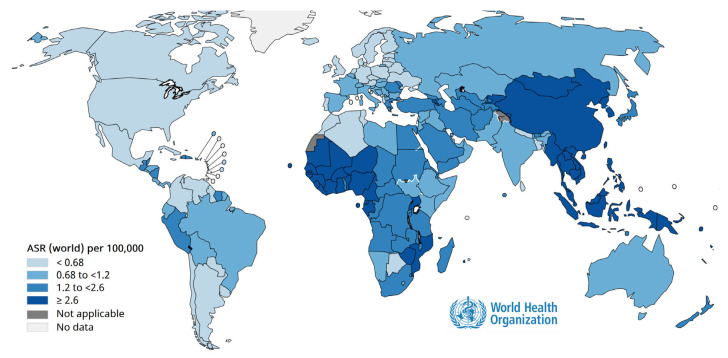
Global burden of hepatitis B–attributable liver cancer and the role of vaccination in prevention. Age-standardized incidence rates of liver cancer attributable to the hepatitis B virus worldwide in 2020, according to the International Agency for Research on Cancer (IARC–WHO). The figure illustrates substantial regional variation linked to historical vaccination coverage and perinatal transmission patterns. Source: IARC Global Cancer Observatory: https://gco.iarc.who.int/causes/infections/tools-map?mode=1&sex=0&continent=0&agent=8&cancer=0&key=asr&scale=quantile (accessed on 1 December 2025).

Cancer prevention through vaccination is one of the most remarkable achievements of modern medicine. Persistent infection with oncogenic viruses such as human papillomavirus (HPV) (Figure 2) and hepatitis B virus (HBV) (Figure 3) is responsible for a large proportion of cervical, anogenital, and hepatocellular carcinomas worldwide [84]. Vaccination against these viruses not only prevents infection but also interrupts the cascade of cellular changes leading to malignancy [84]. As shown in Figure 2 and Figure 3, population-based programs have demonstrated substantial declines in cancer incidence in areas with high vaccination coverage, underscoring vaccines as powerful tools for primary cancer prevention and essential components of comprehensive public health strategies [85]. To illustrate the global impact of oncogenic viral infections and the preventive potential of vaccination, Figure 2 presents the geographic distribution of cancers attributable to human papillomavirus.

The marked geographic heterogeneity reflects differences in vaccine coverage, screening programs, healthcare access, and socioeconomic conditions, underscoring the importance of strengthening HPV vaccination strategies in high-burden regions.

To further illustrate the long-term oncological consequences of chronic viral infections and the preventive impact of vaccination, Figure 3 depicts the worldwide distribution of liver cancers attributable to the hepatitis B virus.

High-incidence regions largely overlap with areas of historical low birth-dose coverage and limited access to early-life vaccination, emphasizing the central role of timely HBV immunization in reducing future liver cancer burden.

#### 2.3.1. HPV

Human papillomavirus (HPV) infection is among the most common viral infections worldwide. It represents the leading cause of cervical cancer, as well as a substantial proportion of anogenital and oropharyngeal cancers. More than 200 HPV genotypes have been identified, of which at least 14 are considered high-risk due to their oncogenic potential. Persistent infection with these high-risk strains, particularly HPV 16 and 18, is responsible for nearly 70% of cervical cancer cases globally [86]. The burden of HPV-related disease underscores the importance of prevention through vaccination as a primary public health strategy [87].

HPV vaccines represent a landmark achievement in cancer prevention. The first-generation vaccines targeted HPV types 16 and 18, while subsequent formulations expanded their coverage to include types 6 and 11 (which cause most genital warts) and additional oncogenic types such as 31, 33, 45, 52, and 58. The nonavalent vaccine currently in use provides the broadest protection, covering approximately 90% of HPV types associated with cervical cancer and offering significant protection against other HPV-related malignancies [88].

The effectiveness of HPV vaccination has been demonstrated in multiple populations worldwide. As reflected in Figure 2, countries with high coverage rates have reported dramatic reductions in HPV prevalence, precancerous lesions, and cervical cancer incidence among young women. In addition, herd immunity benefits have been observed, with declines in genital warts and HPV-related disease among unvaccinated individuals, including men. These outcomes highlight the potential of vaccination not only to protect individuals but also to shift population-level disease dynamics, moving toward the elimination of cervical cancer as a public health problem [89].

Vaccination is most effective when administered before the onset of sexual activity, typically during early adolescence. However, evidence supports extending vaccination to older adolescents and young adults, as many remain unexposed to high-risk HPV strains. Increasingly, public health strategies also emphasize vaccination in boys and men. While cervical cancer prevention remains the primary goal, vaccinating males broadens protection against anal, penile, and oropharyngeal cancers and further strengthens herd immunity [3,85,89].

Despite the proven effectiveness of HPV vaccination, significant challenges remain. Global coverage is uneven, with wide disparities between high-income and low- and middle-income countries. Barriers include limited resources, lack of awareness, social and cultural resistance, and logistical difficulties in delivering vaccines to adolescents. Addressing these challenges requires concerted international efforts, including support for vaccine affordability, education campaigns to build public confidence, and the integration of HPV vaccination into broader reproductive and adolescent health programs [84,87,88].

HPV vaccination is not a replacement for screening but complements it. Cervical cancer screening remains essential, particularly in older women who may not have been vaccinated. The integration of vaccination with screening programs offers a comprehensive approach that maximizes prevention, ensuring both immediate and long-term reductions in HPV-related disease burden [3,85,87,89].

In summary, HPV vaccination is a transformative intervention that directly prevents infection with oncogenic HPV types, reduces the incidence of precancerous lesions, and ultimately decreases cancer risk. By extending coverage to both sexes and ensuring equitable access worldwide, HPV vaccination has the potential to eliminate cervical cancer and significantly reduce the global burden of HPV-associated malignancies [3,84,85,87,88,89].

#### 2.3.2. Hepatitis B

Hepatitis B virus (HBV) infection remains one of the most important causes of chronic liver disease and cancer worldwide. It is estimated that nearly 300 million people are living with chronic HBV infection, with more than 800,000 deaths each year attributed to complications such as cirrhosis and hepatocellular carcinoma. HBV is a highly transmissible virus, spreading through blood and bodily fluids, and infection acquired early in life is particularly concerning due to its high likelihood of progressing to chronic disease. Infants who contract HBV infection perinatally have a 90% risk of developing chronic infection, compared to less than 10% in adults. This underscores the importance of early prevention through vaccination as a crucial public health priority [36].

The introduction of the hepatitis B vaccination has transformed the epidemiology of HBV. Universal vaccination programs, particularly those including a birth dose, have drastically reduced HBV prevalence in children and young adults. In countries that implemented widespread vaccination in the 1980s and 1990s, the rates of chronic infection in children under five have dropped to less than 1%. Perhaps most importantly, long-term studies have shown substantial declines in the incidence of hepatocellular carcinoma among vaccinated cohorts, making HBV vaccination one of the most successful examples of cancer prevention through vaccination [36,37,43].

The hepatitis B vaccine is a recombinant subunit vaccine containing the hepatitis B surface antigen (HBsAg). It induces protective antibody responses in more than 95% of healthy infants, children, and young adults. Protection is long-lasting, with immunity persisting for at least 30 years in most individuals, and booster doses are generally not required for immunocompetent persons. For optimal impact, the World Health Organization recommends a birth dose within 24 h of delivery, followed by completion of the primary vaccine series. This strategy effectively interrupts mother-to-child transmission, which is the predominant route of infection in endemic regions [5,31,38,42].

Beyond preventing perinatal and early childhood infection, hepatitis B vaccination protects individuals later in life from sexual, injection-related, and occupational exposure. It is vital for healthcare workers, immunocompromised patients, and people in high-endemicity regions. Adolescent and adult vaccination complements infant programs by closing immunity gaps and reducing transmission [5,31,36,37,38,42,43].

Despite remarkable progress, challenges remain in achieving universal HBV control. Global coverage with the three-dose infant series has reached approximately 80%, whereas birth-dose coverage remains at around 50%, with significant regional disparities. Barriers include home births without immediate access to healthcare, limited cold-chain capacity, and insufficient awareness of the importance of the birth dose. Expanding timely access, particularly in low-resource settings, is critical to eliminating HBV transmission [90].

The long-term benefits of HBV vaccination extend far beyond infection control. By preventing chronic hepatitis and liver cancer, vaccination reduces healthcare costs, improves quality of life, and lessens the socioeconomic burden on families and health systems (Table 3). In the context of global cancer prevention, hepatitis B vaccination stands as a model of success, demonstrating how targeted vaccination programs can achieve measurable reductions in cancer incidence and mortality [31,43,90].

In summary, hepatitis B vaccination is a cornerstone of global health and cancer prevention. It protects individuals from one of the most lethal infectious carcinogens, prevents mother-to-child transmission, reduces chronic liver disease (Table 3), and has been proven to lower hepatocellular carcinoma incidence at the population level. Achieving universal access to timely vaccination, especially the birth dose, is essential for progressing toward the elimination of HBV as a public health threat and securing healthier futures for generations to come [31,36,42,43,90].

### 2.4. Impact on Cardiovascular Diseases and Other Comorbidities

The relationship among infectious diseases, inflammation, and chronic conditions, including cardiovascular disease, has gained increasing recognition in recent decades. Infections act as acute stressors that can destabilize vulnerable physiological systems, particularly in older adults or individuals with comorbidities. Vaccination, by preventing these infections, reduces the incidence of acute illness and has been associated with a lower risk of secondary complications, thereby potentially contributing to healthier aging and reduced mortality [46,61]. To contextualize the long-term clinical consequences of hepatitis B infection and the preventive value of early vaccination, Table 3 summarizes the risk of chronic infection according to age at exposure and host characteristics.

As shown in Table 3, the probability of progression to chronic hepatitis B is strongly dependent on age at infection, with the highest risk occurring following perinatal and early childhood exposure. These data explain the disproportionate burden of chronic liver disease and hepatocellular carcinoma observed in regions with historically low birth-dose coverage and underscore the critical importance of timely neonatal vaccination for long-term cancer prevention.

#### 2.4.1. Vaccination and Cardiovascular Health

Respiratory infections, including influenza and pneumococcal disease, are strongly linked to acute cardiovascular events. The risk of myocardial infarction increases up to sixfold shortly after influenza infection, particularly in older adults and those with coronary disease. Influenza vaccination has been associated with a lower risk in several observational studies, and selected randomized trials have reported fewer major cardiovascular events and deaths after acute myocardial infarction, with meta-analyses showing a 30–36% reduction in myocardial infarction. Consequently, major cardiology societies recommend annual influenza vaccination as part of routine cardiovascular care [46,47,54,61].

Pneumococcal vaccination provides complementary protection by reducing systemic inflammation and the risk of cardiovascular complications associated with pneumonia. Severe pneumococcal infections can trigger vascular endothelial dysfunction, accelerate atherosclerosis, and increase the likelihood of stroke or myocardial infarction. Vaccines that cover a broader spectrum of pneumococcal serotypes, therefore, contribute to cardiovascular prevention alongside their primary role in reducing invasive pneumococcal disease [41,46,47,48,54,61].

#### 2.4.2. Role in Other Chronic Conditions

Beyond cardiovascular health, vaccination also confers protection in individuals with chronic diseases, including diabetes, chronic respiratory conditions, and renal disease. People with diabetes, for example, are two to six times more likely to experience complications or die from influenza compared to the general population. Vaccination in this group has been associated with reduced hospitalizations, pneumonia, and cardiovascular events in several observational and interventional studies, ultimately leading to a lower all-cause mortality rate. Similarly, patients with chronic obstructive pulmonary disease or asthma experience fewer exacerbations and reduced hospital admissions when protected against influenza and pneumococcal infections [18,54,82,91].

Emerging evidence also indicates that vaccination reduces the systemic inflammatory burden that drives many chronic diseases. Chronic low-grade inflammation—often termed “inflammaging”—is linked not only to cardiovascular disease but also to neurodegenerative and autoimmune conditions. By preventing infections that exacerbate inflammatory cascades, vaccines help preserve immune resilience and mitigate the progression of these comorbidities [92].

#### 2.4.3. COVID-19 and Post-Infectious Complications

The COVID-19 pandemic highlighted the complex interplay between infection and chronic disease. SARS-CoV-2 infection significantly increases the risk of long-term cardiovascular outcomes, including myocardial infarction, thromboembolic events, and heart failure, even months after recovery. Vaccination has been shown to reduce both acute severe outcomes and the risk of post-infectious complications (e.g., long COVID), underscoring the broader cardiovascular benefits of vaccination [93]. Vaccination reduces the risk of developing long COVID. These benefits have been observed in multiple international studies [94,95].

#### 2.4.4. Toward Integrated Prevention

Vaccination should be recognized not only as an intervention for infectious diseases but also as a preventive strategy for chronic non-communicable diseases. By potentially lowering systemic inflammation, reducing hospitalization, and limiting decompensation of underlying conditions, vaccines align with strategies aimed at improving population longevity and quality of life. The integration of adult vaccination into cardiovascular and chronic disease management is, therefore, a public health priority [96].

### 2.5. Long-Term Benefits of Vaccination

Vaccines are often viewed through the lens of their immediate benefits, including protection against infection and reduced transmission. However, growing evidence highlights their long-term impact on health, longevity, and functional capacity. These late effects extend well beyond the prevention of acute illness, shaping trajectories of chronic disease, quality of life, and sustainable aging [97].

One of the central mechanisms underlying these benefits is the modulation of chronic inflammation. With advancing age, the immune system often enters a state of low-grade, persistent activation known as inflammaging. This process contributes to the development of chronic conditions such as diabetes, cardiovascular disease, arthritis, and neurodegenerative disorders. By preventing infections that exacerbate systemic inflammation, vaccines help limit inflammatory burden and reduce the acceleration of age-related decline. Some vaccines may also enhance anti-inflammatory pathways, supporting immune regulation in older adults [18,46,47,48,92]. Building on this biological framework, the concept of “immunofitness” has recently been proposed as a practical objective of healthy aging [98], referring to the immune system’s capacity to maintain resilience, adapt to cumulative stressors, and restore homeostasis after infectious or inflammatory challenges. From this perspective, vaccination contributes to immunofitness not only through pathogen-specific protection but also by strengthening adaptive immune memory, leveraging adjuvanted responses, and potentially inducing trained immunity. Integrating vaccination into broader preventive strategies across the lifespan may therefore support sustained immune resilience and functional health in older adults [98].

Emerging evidence also suggests that vaccination may be associated with a reduced risk of cognitive decline and dementia. Large observational studies have reported lower incidence of Alzheimer’s disease and related dementias among vaccinated individuals, particularly following influenza, herpes zoster, and COVID-19 vaccination. These associations are biologically plausible, given the role of systemic inflammation, vascular injury, and recurrent infections in neurodegenerative processes. However, most available data are observational, and causal relationships remain to be confirmed in prospective and mechanistic studies [99,100].

The benefits are not only biological but also functional. Infections in older adults frequently lead to long-term dependency, reduced mobility, and increased need for institutional care. By preventing these infections, vaccines preserve independence, decrease demand for long-term care services, and protect families and societies from the economic and emotional strain of chronic disability [52].

There is also clear evidence of vaccines preventing the long-term sequelae of infection that directly cause cancer and cardiovascular disease. Hepatitis B and human papillomavirus vaccines reduce the risk of hepatocellular carcinoma and cervical cancer, respectively, providing a powerful demonstration of how vaccination translates into cancer prevention. Similarly, preventing influenza, pneumococcal disease, and herpes zoster reduces cardiovascular complications such as myocardial infarction and stroke, conditions that frequently follow severe infections and contribute substantially to morbidity in older populations [3,46,70].

Beyond health outcomes, vaccination has broad, long-term societal and economic effects. By reducing chronic disease and disability, vaccines lower healthcare costs and enhance the sustainability of health systems. This creates space to allocate resources to other pressing needs while simultaneously promoting healthier, more productive aging populations [36,43,52].

Vaccines are not solely a defense against immediate infection; they are long-term investments in human health. By reducing chronic inflammation, preventing secondary complications, and lowering the risk of cancers and cardiovascular disease, vaccines contribute to healthier aging and extended life expectancy. These late effects underscore the need to adopt a life-course approach to vaccination, ensuring protection across all ages to maximize both individual and societal benefits [101].

## 3. Proposed Immunization Schemes

A life-course approach to vaccination has become a central principle in modern public health (Figure 1). Proposed schemes integrate vaccination across childhood, adolescence, adulthood, and older age to maximize protection and minimize gaps. Early-life schedules include hexavalent and updated pneumococcal conjugate vaccines, complemented by maternal vaccination. Adolescents benefit from HPV vaccination and booster doses of Tdap and meningococcal vaccines. In adults, annual influenza and COVID-19 boosters, pneumococcal conjugate vaccines, zoster vaccines, and hepatitis vaccines are recommended based on individual risk. For older adults, high-dose or adjuvanted influenza vaccines, along with RSV and zoster vaccines, address immunosenescence. Overall, these schemes represent a shift toward continuous, life-course protection that supports healthy aging and reduces morbidity (Figure 1).

## 4. Limitations

The second part of this review has significant limitations. It is a narrative, perspective-based synthesis without a systematic search strategy or formal quality assessment, resulting in variable evidence strength across topics. While vaccine effectiveness in preventing infections, hospitalizations, and mortality is well established, evidence of broader benefits, such as reduced cardiovascular events, mitigation of comorbidities and functional decline, or preservation of independence, largely comes from observational studies and biological plausibility and should be viewed as associative rather than causal.

With respect to cardiovascular outcomes, it is important to distinguish evidence from randomized controlled trials from that from observational studies. While selected randomized trials and meta-analyses have reported reductions in major adverse cardiovascular events following influenza vaccination in high-risk populations, much of the broader literature is based on observational designs. These studies are susceptible to residual confounding, healthy-user effects, and differences in healthcare access and comorbidity profiles. Moreover, not all investigations have reported consistent protective effects, and heterogeneity in study populations, outcome definitions, and follow-up periods contributes to variability in findings. Therefore, the current evidence supports an association between vaccination and improved cardiovascular outcomes in selected contexts, but does not provide definitive proof of a generalized cardioprotective effect.

Much of the available evidence derives from high-income countries, limiting generalizability to Latin America and the Caribbean, where vaccination coverage, health systems, and disease burden vary widely. The vaccination schemes proposed are conceptual frameworks, not prescriptive recommendations, and should be adapted to local contexts and national priorities. As with all narrative reviews, publication bias cannot be excluded, and emerging findings should be interpreted cautiously.

In addition, the successful implementation of life-course vaccination strategies is constrained by persistent structural and behavioral barriers. Vaccine hesitancy, driven by misinformation, mistrust in institutions, cultural beliefs, and previous negative healthcare experiences, continues to limit uptake in many settings. Inequities in access related to socioeconomic status, geographic isolation, migration, and fragile health systems further exacerbate coverage gaps, particularly in low- and middle-income countries. Policy-related challenges, including fragmented financing mechanisms, competing public health priorities, limited adult immunization infrastructure, and insufficient integration into chronic disease management, also hinder the systematic adoption of lifelong vaccination approaches. Addressing these barriers will require coordinated efforts involving public engagement, health system strengthening, sustainable financing, and evidence-informed policymaking.

Declining public confidence in vaccines and the spread of misinformation may further limit the real-world impact of vaccination programs, even where effective vaccines are available.

While the review aims to provide a global overview, its emphasis on Latin America and the Caribbean may limit its direct applicability to other regions with different health system structures and policy environments.

Furthermore, the available evidence on vaccine efficacy, effectiveness, and long-term outcomes is disproportionately derived from high-income countries with well-resourced health systems and mature immunization infrastructures. Data from Latin America and the Caribbean, as well as from other low- and middle-income regions, remain comparatively limited and heterogeneous. Differences in epidemiological profiles, healthcare access, cold-chain capacity, workforce availability, and programmatic implementation may influence real-world vaccine performance and coverage in these settings. Consequently, extrapolating findings across regions should be undertaken cautiously. Future research should prioritize region-specific randomized trials, implementation studies, and longitudinal cohorts to better characterize geographical variations in vaccine impact and to inform context-appropriate vaccination policies.

Unfortunately, many observational studies assessing broader vaccine-related benefits may be affected by selection bias and healthy-user effects, in which individuals who choose to be vaccinated tend to have better baseline health status, greater healthcare engagement, and more favorable socioeconomic conditions. These factors may partially account for observed associations between vaccination and reduced morbidity or mortality, independent of direct biological effects. Although statistical adjustment is commonly applied, residual confounding factors related to health-seeking behavior and access to care cannot be fully excluded.

The cost-effectiveness and operational feasibility of comprehensive life-course vaccination strategies may vary substantially across health systems and socioeconomic contexts. While several adult and older-adult vaccination programs have demonstrated favorable economic profiles in high-income settings, evidence remains limited for many low- and middle-income countries. Constraints related to financing, workforce capacity, cold-chain infrastructure, competing health priorities, and long-term sustainability may affect large-scale implementation. Further context-specific economic evaluations and implementation studies are needed to inform resource allocation and optimize program design.

Finally, the predominance of observational data underscores the need for well-designed randomized controlled trials and large-scale longitudinal cohort studies to more robustly evaluate long-term vaccine effects, clarify causal relationships, and quantify their impact on healthspan, functional outcomes, and chronic disease trajectories.

## 5. Conclusions

Vaccination is one of the most transformative public health achievements, with benefits extending well beyond the prevention of acute infectious diseases. As shown in this review, vaccines not only save lives early in life and prevent severe illness at all ages but also contribute to cancer prevention, are associated with reduced cardiovascular complications, and mitigate comorbidities, with long-term benefits for healthy aging, reduced disability, and improved quality of life, primarily supported by observational evidence. In addition, emerging data suggest that vaccination may protect against cognitive decline and dementia, further supporting its role in promoting healthy aging.

Achieving sustained, life-course vaccination coverage also requires strong interdisciplinary collaboration. Effective responses to vaccine hesitancy and access barriers depend on coordinated efforts among immunologists, clinicians, public health professionals, social scientists, communication specialists, and policymakers. Integrating scientific evidence with behavioral insights, community engagement strategies, and supportive regulatory frameworks is essential for building public trust, improving risk communication, and ensuring the successful implementation of vaccination programs across diverse populations.

Part 1 outlined the biological basis of immunosenescence, trained immunity, and vaccine-mediated immune modulation across the lifespan, while Part 2 emphasized vaccines as tools to extend life and healthspan, prevent chronic sequelae, and enhance societal resilience. Together, these findings reinforce vaccination as a life-course strategy rather than a childhood-only intervention.

Future research agendas should prioritize randomized controlled trials and long-term prospective studies specifically designed to assess the sustained clinical, functional, and socioeconomic benefits of vaccination across the life course. Strengthening region-specific evidence, particularly in Latin America and the Caribbean, will be essential for developing equitable and effective life-course vaccination strategies. While grounded in the experience of the Americas, many of the principles discussed in this article are applicable to diverse global settings.

Recent declines in vaccine confidence and the resurgence of organized anti-vaccination movements represent a growing threat to the sustainability of immunization achievements, particularly in childhood vaccination programs. Reduced coverage increases the risk of outbreaks of preventable diseases, reverses gains in child survival, and undermines herd immunity. If left unaddressed, these trends may compromise not only short-term disease control but also the long-term health, educational, and economic benefits generated by high vaccination rates. Strengthening evidence-based communication, community engagement, and institutional trust is therefore essential to preserve the societal value of immunization.

Ensuring the long-term impact of life-course vaccination will also require robust economic evaluations and delivery models that are feasible given local health-system capacities.

Looking ahead, vaccine policies must emphasize integration, equity, and innovation. Expanding research, ensuring access across all ages, and embedding vaccination into health system planning are essential to maximizing its contribution to healthier, more resilient populations across generations.

## Figures and Tables

**Table 1:** Efficacy of recombinant zoster vaccine (RZV) against herpes zoster (HZ) and post-herpetic neuralgia (PHN) in key adult and immunocompromised populations, based on randomized clinical trials.

Population	Efficacy Against HZ ^a^	Efficacy Against PHN ^a^	References
Adults ≥50 years ^a^	97.2% (95%CI 93.7–99.0)	91.2% (95%CI 75.9–97.7)	[73,74,75]
Adults ≥70 years ^a^	91.3% (95%CI 86.8–94.5)	88.8% (95%CI 68.7–97.1)	[75]
Immunocompromised ^a,b^	~88%	~86%	[75,76,77,78]
ASTC ^a,d^	68.2% (95%CI 55.6–77.5)	90% (95%CI 22–100) ^c^	[79]
Autoimmune diseases ^a,e^	90.5% (95%CI 73.5–97.5)	Not available	[80]

^a^ Efficacy from clinical trials. ^b^ Includes patients with HIV, transplant recipients, cancer, or autoimmune diseases. ^c^ IRR, 0.1 (95%CI 0.00–0.78). ^d^ ASTC, Autologous Stem Cell Transplantation. ^e^ potential immune-mediated diseases (pIMDs), most commonly psoriasis, spondyloarthropathy, and rheumatoid arthritis.

**Table 2:** Impact of recombinant zoster vaccine (RZV) on quality of life, functional outcomes, and healthcare utilization in selected populations, based on randomized and observational studies.

Population	Key QoL Benefits	Evidence Level
Immunocompromised	83% reduction in HZ-related hospitalizations	Randomized clinical trial subgroup
Adults ≥80 years	91% preservation of independence in activities of daily living (ADLs)	Observational
Chronic pain patients	3.2-fold lower pain exacerbation risk	Registry data

**Table 3:** Risk of chronic hepatitis B infection and long-term clinical outcomes according to age at exposure and host characteristics, based on epidemiological and longitudinal studies.

Age Group at Time of Infection	Risk of Chronic Infection	Possible Clinical Outcome
Newborns (HBV-positive mother)	~90%	Very high likelihood of developing chronic hepatitis
Children under 5 years	~20–30%	Moderate risk of progression to chronic infection
Immunocompetent adults	<5–10%	Most individuals clear the infection spontaneously
Patients on hemodialysis	~40%	Moderate to high risk of chronic infection
Immunocompromised individuals	~20%	Moderate risk of chronic infection

## Data Availability

Not applicable.
